# Dosing oral-liquid azithromycin suspensions: do users follow instructions?

**DOI:** 10.1080/07853890.2021.1896105

**Published:** 2021-09-28

**Authors:** Inês Neves, M. Deolinda Auxtero

**Affiliations:** ^a^PharmaSci Lab, Centro de Investigação Interdisciplinar Egas Moniz (CiiEM), Egas Moniz Cooperativa de Ensino Superior, Caparica, Portugal

## Abstract

**Introduction:**

Oral-liquid suspensions are pharmaceutical forms usually accompanied by dosing devices [1]. The type of device influences dose measurement, which is associated with administration errors, with possible impact in the pharmacological treatment [2,3]. On the other hand, as liquid disperse systems, it is essential to shake them before use, to ensure that the active ingredient is well dispersed throughout the vehicle before administration. The aim of this work is to evaluate the degree of awareness of pharmacy students, regarding two key factors for an accurate dose administration: "shake before use", and the choice of an adequate dose-measuring device, packaged with the antibiotic, based on the dosing volume.

**Materials and methods:**

A pilot study was conducted at Instituto Universitário Egas Moniz, with 40 randomly selected pharmacy students with an average age of 21.5 years (± 3.2), who signed an informed consent. Two oral extemporaneously compounded suspensions from azithromycin were used (Azithromycin Baldacci and Zithromax; 40 mg/mL, both including a dosing spoon, an oral syringe and a reconstitution cup). The volunteers were requested to rank the three types of device according to their preference, for doses of 2.5 and 5 mL. It was also recorded if the suspension was shaken before measurement. Otherwise, volunteers were reminded of the importance of the procedure, as stated on product label. The protocol was approved in Egas Moniz, ethics committee.

**Results:**

70% (*n* = 28) of the volunteers chose the dosing device regardless of the dose volume. 50% (*n* = 20) preferred the dosing spoon, whereas 35% (*n* = 14) choose the oral syringe and 15% (*n* = 6) the reconstitution cup as a first choice for administration of the medicines. The main order of preference of the participants was spoon-cup-syringe (*n* = 14, 35%), followed by the syringe-spoon-cup (*n* = 9, 22.5%) and finally spoon-syringe-cup (*n* = 6, 15%). For the participants that questioned about the volume to be measured (*n* = 12, 30%), the cup was never the first option, and the most prevalent sequences were spoon-syringe-cup and syringe-cup-spoon, both with 42% (*n* = 5). In the first measurement, 80% (*n* = 32) of participants did not shake the suspension. After being reminded, the value decreased to 37.8% (*n* = 15), in the second antibiotic.

**Discussion and conclusions:**

The dosing spoon was the preferred device for most participants. The reconstitution cup was mistaken for a measuring device by all participants. Therefore, the inclusion of this type of device may cause gross dosing errors. The "shake before use" instruction which accompanies the label on suspensions, was easily overlooked, despite the specific academic training of these students. This should not be used as the only means of communicating such important information. Upon dispensing of suspensions the pharmacist should make sure that patients fully understand the need to shake the medication well before each administration and are able to choose, and properly use, the most adequate dosing device.
